# Clinical outcomes and characteristics of critically ill patients with influenza- and COVID-19-induced ARDS: A retrospective, matched cohort study

**DOI:** 10.3389/fmed.2022.1027984

**Published:** 2023-01-05

**Authors:** Lev Volkov, Marion Delpuech, Marie Conrad, Guilhem Courte, Aurélie Cravoisy, Lionel Nace, Cedric Baumann, Sébastien Gibot

**Affiliations:** ^1^Service de Réanimation Médicale, Hôpital Central, Centre Hospitalier Régional Universitaire (CHRU), Nancy, France; ^2^Service de Réanimation Médico-Chirurgicale, Centre Hospitalier-Le Mans, Le Mans, France; ^3^Délégation à la Recherche Clinique et à l'Innovation (DRCI), Méthodologie Promotion Investigation (MPI) Department, Methodology, Data Management, and Statistic Unit, University Hospital of Nancy, Vandœuvre-Lès-Nancy, France

**Keywords:** ARDS, influenza, COVID-19, intensive care unit (ICU), mechanical ventilation

## Abstract

**Introduction:**

Seasonal epidemic influenza and SARS-CoV-2 are the most frequent viruses causing acute respiratory distress syndrome (ARDS). To what extent these two etiologies differ in ICU patients remains uncertain. We, therefore, aimed at comparing the severity and outcomes of influenza and SARS-CoV-2-induced ARDS in mechanically ventilated patients.

**Methods:**

This retrospective, analytic, single-center study was conducted in the medical ICU of Nancy University Hospital in France. Adult patients hospitalized with confirmed influenza (from 2009 to 2019) or SARS-CoV-2-induced ARDS (between March 2020 and May 2021) and those under mechanical ventilation were included. Each patient with influenza was matched with two patients with COVID-19, with the same severity of ARDS. The primary endpoint was death in ICU on day 28. The secondary endpoints were the duration of vasopressors, the use of renal replacement therapy, the duration of mechanical ventilation, and the ICU length of stay.

**Results:**

A total of 42 patients with influenza were matched with 84 patients with COVID-19. They had similar sex distribution, age, Charlson comorbidity index, and ARDS severity. On day 28, 11 (26.2%) patients in the influenza group and nine (10.7%) patients in the COVID-19 group had died (*p* = 0.0084, HR = 3.31, CI 95% [1.36–8.06]). In the univariate Cox model, being infected with SARS-CoV-2, SOFA and SAPS II scores, initial arterial pH, PaCO2, PaO2/FiO2, serum lactate level, platelet count, and use of renal replacement therapy were significantly associated with mortality. In the multivariate Cox model, the SOFA score at admission (*p* < 0.01, HR = 1.284, CI 95% [1.081; 1.525]) and the initial pH (*p* < 0.01, HR = 0.618, CI 95% [0.461; 0.828]) were the only predictors of mortality. The type of virus had no influence on mortality, though patients with COVID-19 underwent longer mechanical ventilation and received more neuromuscular blockers and prone positioning.

**Conclusion:**

In mechanically ventilated patients with ARDS, 28-day mortality was higher among patients with influenza as compared to patients with COVID-19 because of a higher initial extra-pulmonary severity. However, the type of virus was not, by itself, correlated with mortality.

## Introduction

The seasonal influenza virus is well known for causing winter epidemics and even unpredictable pandemics. By invading the lower respiratory tract, it can cause acute respiratory distress syndrome (ARDS) and is responsible each year for admissions to the intensive care unit (ICU) with prolonged hospitalization (1, 2). The outbreak of the coronavirus disease 2019 (COVID-19) pandemic overwhelmed hospitals with a great number of patients presenting acute respiratory failure, with many developing ARDS (3). Nowadays, influenza and severe acute respiratory syndrome coronavirus 2 (SARS-CoV-2) are the most frequent viruses causing ARDS (4). Their modes of transmission and clinical presentation are similar. They can cause acute respiratory disease, as well as extra-pulmonary disorders such as cardiac and kidney failure, and endotheliopathy (1, 5, 6). To understand the particularities of COVID-19 in critically ill patients, patients with influenza and COVID-19 have been compared in the literature. However, studies often compared COVID-19 with other multiple causes of ARDS, including viral and bacterial infections (7, 8). Other studies did not focus on critically ill patients (4, 9), and in those that did, not all the patients were under mechanical ventilation (10, 11). Indeed, studies dealing only with mechanically ventilated patients with ARDS are very scarce (12, 13).

This study aimed to compare the characteristics and outcomes of mechanically ventilated ICU patients suffering from influenza or SARS-CoV-2 ARDS of similar pulmonary severity to untangle the influence of the virus type by itself.

## Methods

### Study design and setting

We conducted a retrospective, single-center, and analytical matched cohort study in the ICU, Réanimation Médicale, Hôpital Central of the Nancy Regional and University Hospital Center in France. The study was registered on www.clinicaltrials.gov under the number NCT04941092 and was approved by the Ethics Committee of our University Hospital.

### Participants

The electronic medical database of the hospital was searched for patients hospitalized in our ICU between 2009 and 2019 with the main diagnosis containing “influenza,” and between March 2020 and May 2021 with the main diagnosis containing “COVID-19” or “SARS-CoV-2.” Inclusion criteria were participants older than 18 years, a diagnosis of ARDS according to the Berlin definition criteria (14), influenza or SARS-CoV-2 infection confirmed by reverse transcription-polymerase chain reaction (RT-PCR), and the use of invasive mechanical ventilation. Exclusion criteria were pregnancy, the use of invasive mechanical ventilation for more than 48 h before admission into the ICU, or a secondary transfer to another ICU. Each patient with influenza was matched with two patients with COVID-19, based on the severity of ARDS: severe ARDS or mild to moderate ARDS.

### Endpoints

The primary endpoint was mortality in the ICU within 28 days. The secondary endpoints were the duration of vasopressors, the use of renal replacement therapy, the duration of mechanical ventilation, the length of stay in the ICU, and the duration and amount of sedation.

### Data collection

Data were collected from the patient's medical records and anonymized. Data included baseline demographic characteristics, such as age, sex, height, and weight, patient's past medical history, laboratory findings, and clinico-biological parameters: simplified acute physiology score (SAPS II) and sequential organ failure assessment (SOFA) score, the worst ratio of arterial oxygen partial pressure to fractional inspired oxygen (PaO2/FiO2), highest positive end-expiratory pressure (PEEP) level within the first 24 h of mechanical ventilation, complete blood cells count, blood chemistry, serum lactate concentration, duration of mechanical ventilation, number of prone positioning sessions, use and duration of catecholamines, renal replacement therapy, extracorporeal membrane oxygenation (ECMO), antibiotics, corticosteroids, neuromuscular blockers, and duration and quantity of midazolam, sufentanil, propofol, dexmedetomidine, and chlorpromazine.

### Statistical methods

#### Descriptive and comparative analyses

Baseline characteristics were described as counts and percentages for categorical variables and as median and interquartile ranges for continuous variables. The distribution of the baseline parameters was compared between the two groups of infected patients using an exact chi-square test, exact Fisher test, or Wilcoxon test, according to the type of variable and statistical conditions appliance. The Fisher and chi-square tests were used for qualitative variables. The Fisher test was used in default of the chi-square test when the statistical conditions were not verified. The Wilcoxon test was used for continuous variables as they did not follow a Gaussian distribution.

#### Survival analysis

Patients discharged from the ICU before day 28 were censored at the time of discharge. Associations between each parameter of interest and overall survival measured during the 28 days after ICU admission were assessed by univariate Cox models on paired series. Then, correlations between variables with a *p*-value of <0.05 were estimated by a Pearson coefficient or a Phi coefficient, according to the type of variable. In case of high correlation (r or Phi > 0.75) between variables, a choice was made based on the clinical relevance of the variables of interest. A multivariate Cox model on paired series was then built to assess the association between virus type and mortality within 28 days of ICU admission adjusted for the selected factors. If several models were built, the selection of the final model was made based on the Akaike criterion [the best model being the one with the lowest AIC (Akaike information criterion)]. These analyses were completed by Kaplan–Meier overall survival curves compared using a log-rank test. We estimated the hazard ratios with a confidence interval of 95% and fixed the alpha's risk threshold to 5%. The analyses were performed using SAS version 9.4 (SAS Institute, Inc., Cary, NC, USA).

## Results

### Characteristics of the patients

**Figure 1:**
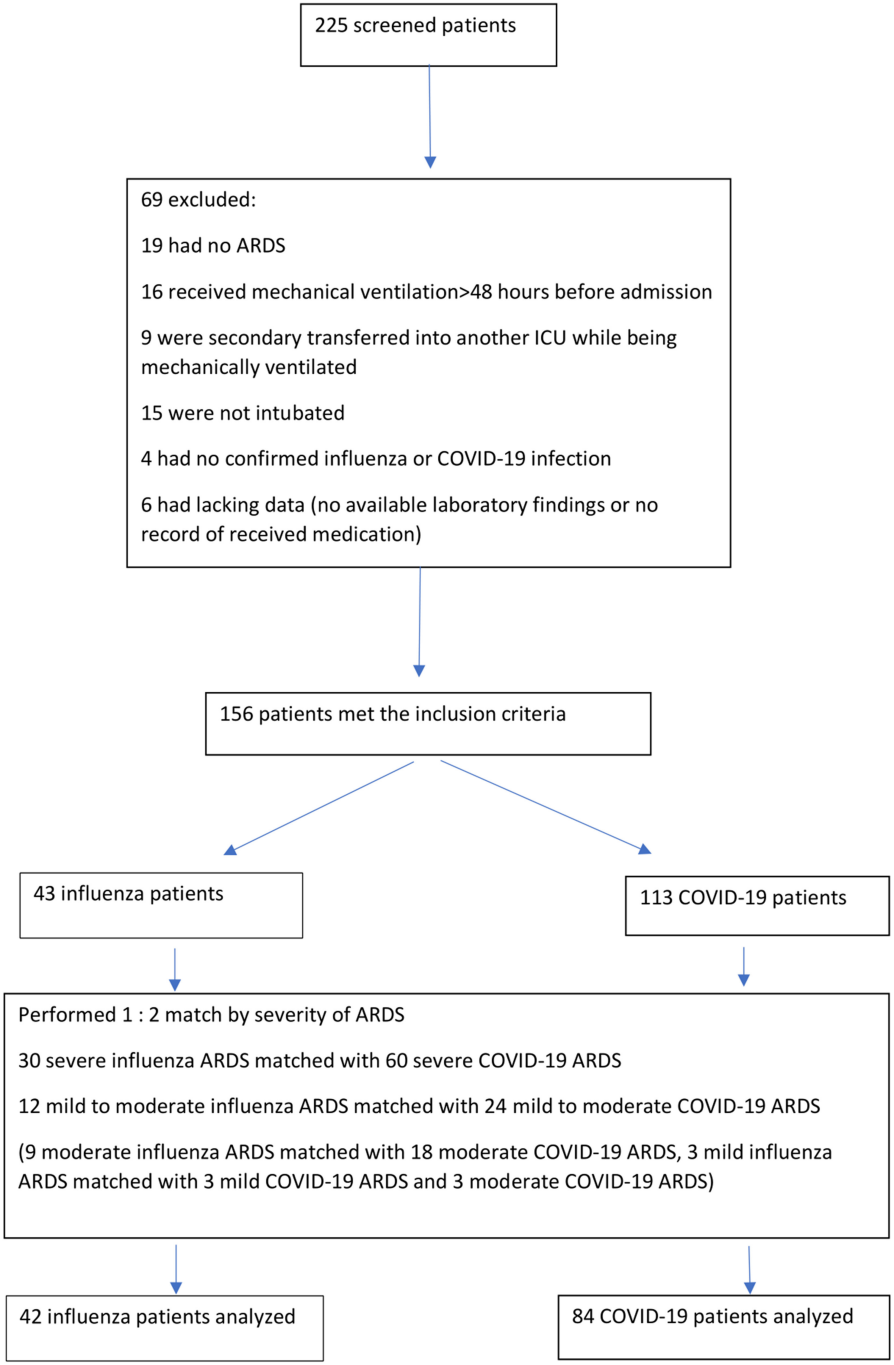
Flow chart.

Among 43 patients with influenza and 113 patients with COVID-19 who met the inclusion criteria, 42 patients with influenza were matched with 84 patients with COVID-19 (Figure 1). A total of 30 influenza patients with severe ARDS were matched with 60 COVID-19 patients with severe ARDS. Notably, 12 influenza patients with mild to moderate ARDS were matched with 24 COVID-19 patients with mild to moderate ARDS (three patients with influenza having mild ARDS were matched with one patient with COVID-19 having mild ARDS and one patient with COVID-19 having moderate ARDS, respectively, as there were not enough mild COVID-19 ARDS). No significant differences were observed for sex, age, and Charlson comorbidity index (Table 1). There were more hematologic malignancies in the influenza group (5 vs. 1). Compared to the patients with COVID-19, patients with influenza had significantly higher median SOFA and SAPS II scores, a lower initial median arterial pH, a higher initial median PaCO2, a higher median serum lactate level, a lower median platelet count, and a higher median serum creatinine level at admission (Table 1). All patients underwent mechanical ventilation.

**Table 1:** Baseline characteristics.

	**Influenza cohort** ** (*n =* 42)**	**COVID-19 cohort** ** (*n =* 84)**	***p-*value**
Age (years)	60.5 [56–69]	66 [58.5–72]	0.11
Male sex (%)	27 (64.3)	52 (61.9)	1
Body mass index, kg/m^2^	29.3 [24.8–33.7]	30.6 [28–34.7]	0.06
Charlson Comorbidity Index	3 [2–4]	3 [2–4]	0.77
Hypertension *n* (%)	22 (52.4)	45 (53.6)	0.62
Chronic obstructive pulmonary disease *n* (%)	7 (16.7)	12 (14.3)	0.72
Diabetes mellitus *n* (%)	10 (23.8)	26 (31)	0.22
Coronary artery disease *n* (%)	6 (14.3)	13 (15.5)	0.75
Solide cancer (%)	4 (9.5)	4 (4.8)	0.46
Hematologic malignancy *n* (%)	5 (11.9)	1 (1.2)	0.03
SOFA	10 [8–12]	6 [4–8]	<0.001
SAPS II	56.5 [42–76]	44 [34.5–55]	<0.001
Severe ARDS *n* (%)	30 (71.4)	60 (71.4)	
Mild to moderate ARDS *n* (%)	12 (28.6)	24 (28.6)	
PaO2/FiO2	72.6 [65.4–115]	84.9 [70.8–115]	0.61
Arterial pH	7.3 [7.2–7.4]	7.4 [7.3–7.4]	<0.001
pCO2	47.9 [39.4–60.1]	41.8 [37.4–46.2]	<0.001
pO2	69.9 [60.6–85.6]	70.9 [62.4–83.6]	0.26
Bicarbonate (mmol/L)	22.6 [20.1–25.8]	25.5 [22.5–27.4]	0.03
Serum lactate (mmol/L)	1.8 [1.3–3.5]	1.3 [1.−1.6]	<0.001
Lymphocytes (G/L)	0.5 [0.3–0.8]	0.7 [0.4–0.8]	0.95
Platelets (G/L)	153 [118–204]	231 [165–279]	<0.001
Creatinine (mg/dL)	1.26 [0.92–2.25]	0.78 [0.61–1.14]	<0.001
Total bilirubine (mg/dL)	0.5 [0.4–0.85]	0.6 [0.5–0.8]	0.40

Unless stated, data are expressed in the median with interquartile range.

### ICU therapies

As shown in Table 2, patients with COVID-19 underwent significantly longer median mechanical ventilation with higher initial PEEP levels, received neuromuscular blockers more often, and underwent prone positioning more often during their ICU stay than patients with influenza. There was no statistical difference between the proportion of patients in each group receiving vasopressors, although patients with COVID-19 received vasopressors for a longer time than patients with influenza. Patients with influenza had a trend of receiving more renal replacement therapy than patients with COVID-19, without statistical significance (28.6 vs. 14.3%, *p* = 0.07). When comparing sedative agents, patients with COVID-19 received midazolam and sufentanil much longer, with higher doses of midazolam. Two patients with influenza were placed under ECMO, but none were in the COVID-19 group.

**Table 2:** ICU therapies and outcome.

	**Influenza cohort,** ***n =* 42**	**COVID-19 cohort,** ***n =* 84**	***p*-value**
28-day death *n* (%)	11 (26.2)	9 (10.7)	<0.01
Death in ICU *n* (%)	13 (31)	16 (19)	0.09
ICU length of stay	11 [5–19]	20 [14–32]	0.80
Length of mechanical ventilation	9.5 [3–14]	18 [12–28.5]	<0.001
Highest PEEP in the first 24 h	10 [8–14]	14 [12–15]	<0.001
Neuromuscular blockers *n* (%)	33 (78.6)	83 (98.9)	<0.01
Prone positioning *n* (%)	14 (33.3)	61 (72.6)	<0.001
Number of prone positioning sessions during ICU stay, if realized	1.5 [1–2]	3 [1–4]	0.36
Vasopressors *n* (%)	39 (92.9)	73 (86.9)	0.33
Duration of vasopressors, days	2 [1–5], *n =* 39	8 [5–15], *n =* 73	<0.001
Renal replacement therapy *n* (%)	12 (28.6)	12 (14.3)	0.07
Duration of antibiotics (days)	7 [4–10]	10 [5–16]	0.02
Corticosteroids *n* (%)	24 (57.1)	47 (56)	0.89
Dose of corticosteroids (mg equivalent prednisone/kg/day)	0.7 [0.6–1]	1 [0.8–1.2]	0.70
Duration of midazolam (days)	4 [2–6], *n =* 41	14 [9–21], *n =* 84	<0.001
Dose of midazolam (mg/kg/day)	1.1 [0.7–1.7], *n =* 39	3.3 [2.5–4.1], *n =* 84	<0.001
Duration of sufentanil (days)	4 [2–7], *n =* 41	14.5 [10–22.5], *n =* 84	<0.001
Dose of sufentanil (μg/kg/day)	2.8 [1.4–4.1], *n =* 39	3 [2.4–3.9], *n =* 84	0.81
Duration of propofol (days)	1 [1–3], *n =* 13	3 [2–4], *n =* 23	0.37
Dose of propofol (mg/kg/day)	9.3 [7.4–15.9], *n =* 13	4.2 [3–9.2], *n =* 19	0.42

Unless stated, data are expressed in the median with an interquartile range.

### Association between the type of virus and survival

**Figure 2:**
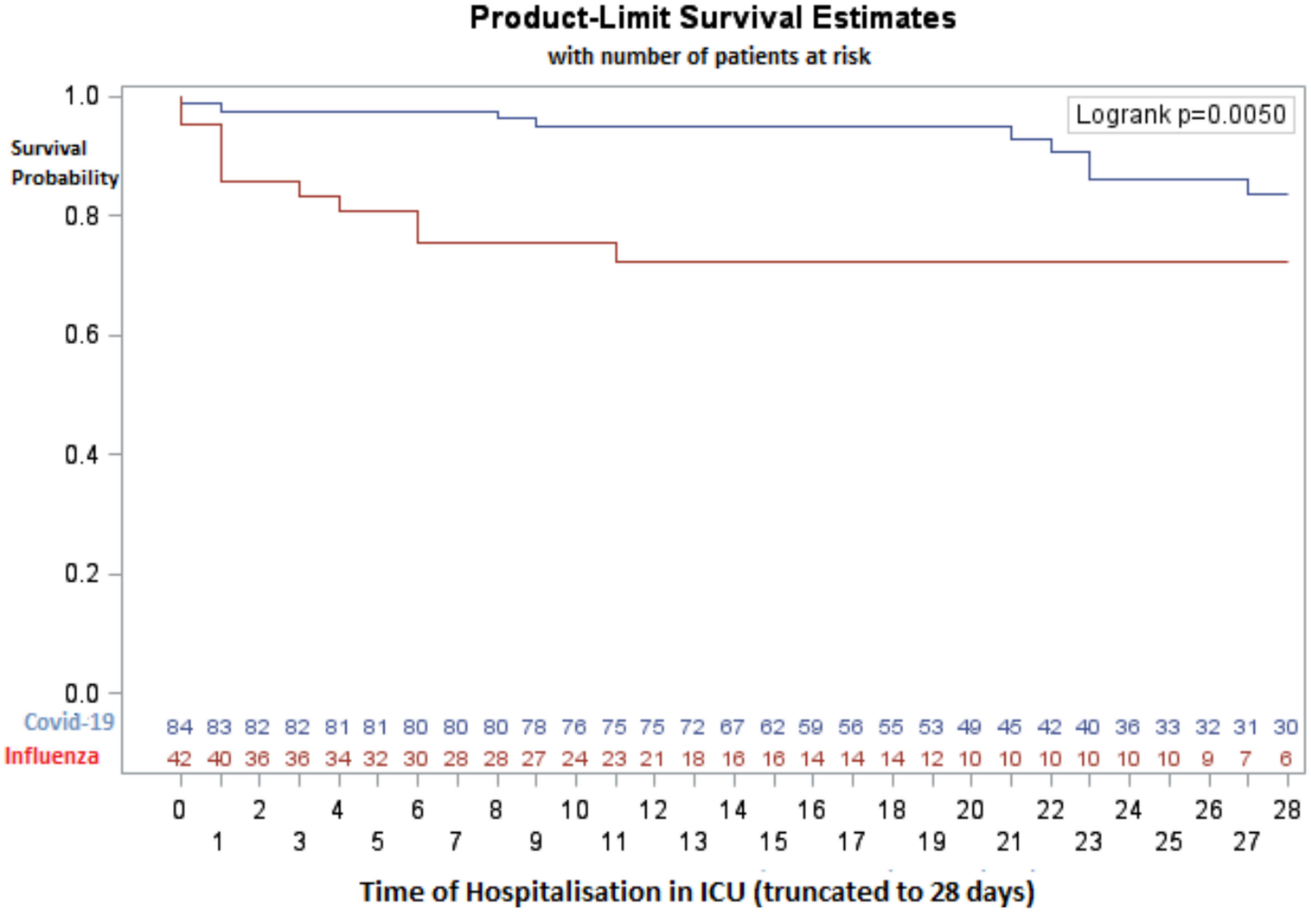
Probability of survival (Kaplan-Meyer curves).

On day 28, 11 (26.2%) patients in the influenza group and nine (10.7%) patients in the COVID-19 group had died (HR = 3.31, CI 95% [1.36–8.06], *p* = 0.0084). Figure 2 shows the Kaplan–Meier curve of the probability of survival from ICU admission to day 28, with a significantly higher probability of survival in the COVID-19 group (*p* = 0.005). Overall, death in ICU was 31% in the influenza group vs. 19% in the COVID-19 group (*p* = 0.09).

The results of univariate Cox models are shown in Table 3. Being infected with influenza, the SOFA and SAPS II scores, initial arterial pH, PaO2/FiO2, PaCO2, serum lactate concentration, platelet count, and use of renal replacement therapy were significantly associated with mortality.

**Table 3:** Predictive factors of mortality (univariate Cox models results).

	**Hazard ratio** ** [95% CI]**	***P* value**
Virus (reference = SARS-CoV-2)	3.3 [1.4; 8.1]	<0.001
SOFA score	1.393 [1.209; 1.606]	<0.001
SAPS II score (categorized, reference ≤ 46.5^*^)	5.197 [1.733; 15.588]	<0.001
Age (years)	1.024 [0.98; 1.071]	0.28
Male gender	1.182 [0.468; 2.985]	0.72
Charlson Comorbidity Index	0.998 [0.819; 1.215]	0.98
Body mass index, kg/m^2^	0.932 [0.849; 1.024]	0.14
Initial arterial pH	0.547 [0.434; 0.690]	<0.05
PaC02 (mmHg)	1.042 [1.017; 1.067]	<0.001
Serum lactate (mmol/L)	1.164 [1.11; 1.221]	<0.001
PaO2/FiO2	0.949 [0.922; 0.977]	<0.001
Platelets (G/L)	0.993 [0.987; 0.998]	0.01
Lymphocytes (G/L)	1.889 [0.77; 4.633]	0.16
Creatinine (mg/dL)	1.026 [0.994; 1.059]	0.11
Total bilirubine (mg/dL)	0.987 [0.874; 1.116]	0.84
Vasopressors	5.146 [0.29; 91.276]	0.26
Renal replacement therapy	4.03 [1.663; 9.762]	<0.01
Neuromuscular blockers	0.412 [0.092; 1.845]	0.24
Corticosteroids	2.048 [0.74; 5.669]	0.16

* 46.5 is the median SAPS II score of the entire cohort.

The analysis of correlation matrices between the variables of interest with a *p*-value of <0.05 in the univariate model and clinical reasoning led to the construction of two multivariate Cox models. Model 1 (Table 4) includes the type of virus, arterial pH, and SOFA score (AIC = 157.7); and model 2 (Table 5) includes the type of virus, arterial pH, and SAPS II score (AIC = 163.9). The first model including SOFA score and pH value showed to be the most parsimonious. The type of virus had no independent effect on mortality.

**Table 4:** Predictive factors of mortality (multivariate Cox model) – SOFA score considered.

	**Hazard ratio [CI 95%]**	***P* value**
Virus (reference=SARS-CoV-2)	0.752 [0.232; 2.443]	0.63
SOFA score	1.284 [1.081; 1.525]	<0.01
Initial arterial pH	0.618 [0.461; 0.828]	<0.01
*AIC*	*157.797*

AIC: Akaike information criterion.

**Table 5:** Predictive factors of mortality (multivariate Cox model) – SAPS II score considered.

	**Hazard ratio [CI 95%]**	***P* value**
Virus (reference = SARS-CoV-2)	1.493 [0.559; 3.988]	0.42
SAPS II score (reference = < 46.5)	2.670 [0.794; 8.980]	0.11
Initial arterial pH	0.638 [0.483; 0.842]	<0.01
*AIC*	*163.883*

AIC: Akaike information criterion.

## Discussion

This analytic retrospective study comparing 42 influenza and 84 COVID-19 mechanically ventilated patients, matched by the severity of ARDS, found that influenza patients had higher 28-day mortality (26.2 vs. 10.7%) (*p* < 0.05). However, multivariate Cox analysis revealed that the type of virus by itself did not affect mortality: SOFA score and initial arterial pH were the only independent predictors of outcome.

When not considering the early studies reporting very high mortality rates that were not confirmed afterward, the mortality of mechanically ventilated patients with COVID-19 in ICU varies between 24 and 43% (3, 15–18), with an important heterogeneity between cohorts (19, 20). The mortality of our patients with COVID-19 is lower despite similar demographics and severity than in other studies. In contrast, the 26.2% mortality rate of our patients with influenza is more consistent with the literature, as reported mortality rates in mechanically ventilated influenza patients with ARDS range between 26 and 45% (21–24). There are no arguments in the literature for a difference in the outcome, length of hospitalization, or mechanical ventilation among the different strains of influenza (25).

Studies comparing influenza and SARS-CoV-2-induced ARDS are scarce. Gjurašin et al. (12) described 42 influenza and 30 COVID-19 intubated patients in a Croatian center with mortality rates of 55 and 63%, respectively. For comparison, the overall ICU mortality in our study was 31% in patients with influenza and 19% in patients with COVID-19. Cobb et al. (10) described 74 patients with influenza and 65 patients with COVID-19 in the medical ICUs of two Washington hospitals, but less than 60% of them were intubated. Furthermore, there were more ARDS cases in their COVID-19 group than in their influenza group, which makes it difficult to compare those patients with ours. Hospital mortality in their ARDS patients was 37% in the influenza group and 46% in the COVID-19 group. Tang et al. (11) described 75 H1N1-induced ARDS from Wuhan and 73 COVID-19-induced ARDS from Beijing. In-hospital mortality was 34.7% among patients with influenza and 28.8% among patients with COVID-19. Here again, the groups had different respiratory severity (PaO2/FiO2 of 107 and 85.8% of mechanically ventilated patients in the influenza group, and 199 and 19.2% in the COVID-19 group). Cárdenas et al. (13), in a single-center study from Mexico, compared 94 influenza and 147 COVID-19 intubated patients with ARDS. Although their patients with influenza had more shock at admission, were more hypoxemic, and had a higher SOFA score, their crude ICU mortality was lower than that of the patients with COVID-19 (22 vs. 39%), which contrasted with our results. Piroth et al. (4) described the characteristics of more than 100000 hospitalized inflluenza and COVID-19 patients, using a large nationwide french database. Among them, 14% were admitted to the ICU. Their in-hospital mortality among mechanically ventilated patients was 26% in the influenza group, and 31.8% in the COVID-19 group. Finally, Ludwig et al. (9) described a German database of 6,762 patients with influenza and 2,343 patients with COVID-19, with 15% admitted to the ICU. Their mortality rate in mechanically ventilated patients was 36% in patients with influenza and 47% in patients with COVID-19. However, only 54% of ventilated patients with COVID-19 and 14% of ventilated patients with influenza had ARDS. It is, therefore, difficult to discuss and compare the mortality of these studies with ours, as they were not all focusing on critically ill patients and described patients with different respiratory severity. Of note, all these studies included patients with COVID-19 at the beginning of the pandemic, with only one study extending to October 2020 (13).

The difference in mortality between patients with influenza and COVID-19 observed in our study seems to be explained by the increased initial severity of patients with influenza, as witnessed by significantly higher SOFA and SAPS II scores at admission. As the patients were matched by the severity of ARDS, the difference in these scores is mainly due to a difference in extra-pulmonary severity. However, patients with influenza had an initial higher PaCO2 level, with lower pH, which could reflect impaired pulmonary compliance. Unfortunately, compliance could not be calculated in our study. Furthermore, significantly lower pH in the first 24 h could be explained by metabolic acidosis due to initial hemodynamic and renal failure. Botta et al. (18) showed a significant association between initial pH and 28-day mortality in their patients with COVID-19. In our study, patients with influenza had more acute kidney injury (AKI) with a higher serum creatinine level at admission, and a trend toward a more frequent use of renal replacement therapy (RRT) (28 vs. 14%) during the ICU stay, though without statistically significant difference. AKI is a well-described complication of COVID-19 (26) and influenza (27), especially in critically ill patients, and is a major risk factor for mortality. In the previously cited studies, AKI in patients with COVID-19 varies between 18 and 58%, with a need for RRT between 11 and 28% (3, 9–12, 16, 18, 19), and is associated with the highest mortality if occurring within the first 15 days (3). AKI in patients with influenza varies between 11 and 59% (9, 12, 22, 25).

In our study, patients with influenza had significantly higher lactate levels on admission and a lower platelet count, which could reflect the initial severity of the shock. By contrast, patients with COVID-19 received vasopressors significantly longer, but this was probably the consequence of a need for longer mechanical ventilation and an increased amount of sedative agents, due to secondary respiratory worsening, rather than primary hemodynamic failure.

Thus, it could be hypothesized that for an equal severity of ARDS, a greater proportion of patients with influenza has multi-organ failure at admission into the ICU. Indeed, our multivariate model shows that initial arterial pH and SOFA score are independent predictors of mortality in our patients. For example, each decrease of 0.1 points in the initial arterial pH increases the risk of mortality by 61%. In this model, there was no statistical association between the type of virus and mortality, meaning that having influenza or SARS-CoV-2 is not, by itself, a risk factor for mortality. In other words, there would be no difference in terms of mortality between patients with influenza and COVID-19, if their initial severity was similar.

Finally, patients with COVID-19 underwent longer mechanical ventilation, with higher initial PEEP levels, and received more neuromuscular blockers and prone position, which is consistent with the literature (8–10, 12). Regarding the use of sedative agents, patients with COVID-19 received midazolam longer and at a higher dose, as previously reported (28, 29). Possible explanations are that COVID-19 had a high respiratory drive and tachyphylaxis due to prolonged mechanical ventilation. Furthermore, challenges in entering patients' rooms due to isolation precautions and fear of self-extubation could lead to higher sedation (29).

Our work has several limitations. First, it is a retrospective, single-center study possibly dampening its external validity. Second, the data of patients with influenza were recorded for a period of 10 years, whereas those of patients with COVID-19 were recorded over 1 year, leading to a comparison of different periods with possible different medical practices. Third, sample sizes were small, leading to a possible misinterpretation of the results. However, to our knowledge, our study is the first to compare critically ill patients with influenza and COVID-19 matched by the severity of respiratory failure. All our patients had ARDS and underwent mechanical ventilation in the same center, making their outcomes comparable, and the results of our study pertinent.

## Conclusion

This study compared mechanically ventilated patients with influenza and COVID-19 admitted to the ICU and matched by the severity of ARDS and found that the mortality was higher among patients with influenza, due to higher extra-pulmonary severity. However, the virus type, by itself, influenza or SARS-CoV-2, was not predictive of mortality.

## Data availability statement

The data analyzed in this study is subject to the following licenses/restrictions: Anonymized data are available upon request by the corresponding author. Requests to access these datasets should be directed to LV, leva.volk.tours@gmail.com.

## Ethics statement

The studies involving human participants were reviewed and approved by Comité d'éthique du CHRU de Nancy. Written informed consent for participation was not required for this study in accordance with the national legislation and the institutional requirements.

## Author contributions

LV, MC, GC, AC, LN, and SG were involved in the management of the patients. LV and SG collected data. CB and MD analyzed data. LV, SG, CB, and MD wrote the manuscript. All authors approved the manuscript.
